# Oridonin: A Review of Its Pharmacology, Pharmacokinetics and Toxicity

**DOI:** 10.3389/fphar.2021.645824

**Published:** 2021-07-05

**Authors:** Xiang Li, Chuan-Tao Zhang, Wei Ma, Xin Xie, Qun Huang

**Affiliations:** ^1^ Department of Ophthalmology, School of Pharmacy, College of Medical Technology, Hospital of Chengdu University of Traditional Chinese Medicine, Chengdu University of Traditional Chinese Medicine, Chengdu, China; ^2^ Department of Respiratory, School of Pharmacy, College of Medical Technology, Hospital of Chengdu University of Traditional Chinese Medicine, Chengdu University of Traditional Chinese Medicine, Chengdu, China

**Keywords:** oridonin, pharmacology, pharmacokinetics, toxicity, *Isodon rubescens* (Hemsl.) H.Hara

## Abstract

Oridonin, as a natural terpenoids found in traditional Chinese herbal medicine *Isodon rubescens* (Hemsl.) H.Hara, is widely present in numerous Chinese medicine preparations. The purpose of this review focuses on providing the latest and comprehensive information on the pharmacology, pharmacokinetics and toxicity of oridonin, to excavate the therapeutic potential and explore promising ways to balance toxicity and efficacy of this natural compound. Information concerning oridonin was systematically collected from the authoritative internet database of PubMed, Elsevier, Web of Science, Wiley Online Library and Europe PMC applying a combination of keywords involving “pharmacology,” “pharmacokinetics,” and “toxicology”. New evidence shows that oridonin possesses a wide range of pharmacological properties, including anticancer, anti-inflammatory, hepatorenal activities as well as cardioprotective protective activities and so on. Although significant advancement has been witnessed in this field, some basic and intricate issues still exist such as the specific mechanism of oridonin against related diseases not being clear. Moreover, several lines of evidence indicated that oridonin may exhibit adverse effects, even toxicity under specific circumstances, which sparked intense debate and concern about security of oridonin. Based on the current progress, future research directions should emphasize on 1) investigating the interrelationship between concentration and pharmacological effects as well as toxicity, 2) reducing pharmacological toxicity, and 3) modifying the structure of oridonin—one of the pivotal approaches to strengthen pharmacological activity and bioavailability. We hope that this review can provide some inspiration for the research of oridonin in the future.

## Introduction

**FIGURE 1:**
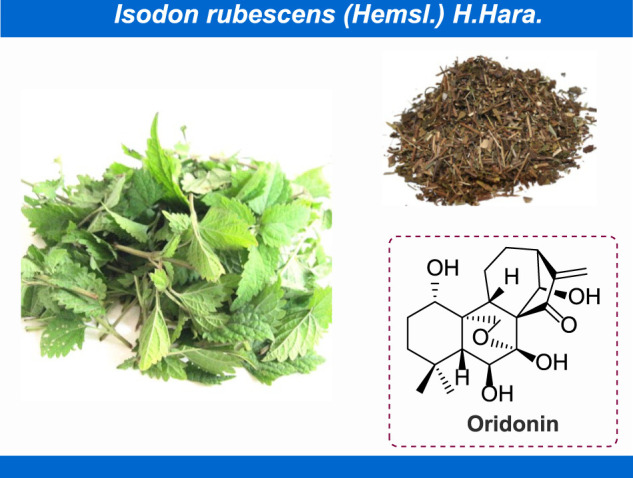
Oridonin isolated from *Isodon rubescens* (Hemsl.) H.Hara.

Oridonin, (PubChem CID: 5321010, CAS No: 28957-04-2, MW: 364.4 g/mol), with the molecular formula of C_20_H_28_O_6_ (Cheng et al., 2019), is a naturally occurring terpenoids that mainly exists in *Isodon rubescens* (Hemsl.) H.Hara (Figure 1; Yang I.-H. et al., 2017; Jian et al., 2019; Meng et al., 2019). In thousands of years of clinical practice, the *Isodon rubescens* (Hemsl.) H.Hara has been widely applied as central agent in classic traditional Chinese medicine (TCM) formulas with its efficacy of clearing away heat and detoxifying, boosting blood circulation and alleviating pain. Generally, *I. rubescens* (Hemsl.) H.Hara is frequently utilized in the treatment of acute and chronic pharyngitis, tonsillitis and bronchitis in clinic (Zhang et al., 2020). As the main bioactive chemical component of *I. rubescens* (Hemsl.) H.Hara, in recent years, numerous achievements have been witnessed on the exploration of pharmacological effects of oridonin, such as anti-inflammatory (Cummins et al., 2019; He et al., 2019), anti-cancer (Vasaturo et al., 2018; Jeon et al., 2019; Hu et al., 2020), anti-microbial (Li D. et al., 2016), anti-sepsis (Zhao et al., 2016), neuroprotection (Lin et al., 2019), immunoregulation (Guo et al., 2013) and so on. Consequently, to some extent, these rapid advancements in the discovery of the pharmacological activity of oridonin have provided extensive opportunities for the development of innovative disease strategies. On the other hand, there have been mounting reports concentrated on the adverse reactions of oridonin. Recent studies have shown that oridonin can cause suicidal erythrocyte death, induce the expression and activation of CYP2C and CYP3A family, and interfere with the early embryonic development of zebrafish. Under this background, thereby motivated, we herein to summarize the latest and comprehensive information on the pharmacology, toxicity and pharmacokinetics of oridonin, to excavate the potential of this natural active ingredient in the treatment of various diseases and furnish basic information for the rational and secure utilization of oridonin.

## Pharmacology

### Anti-Inflammatory Activity

According to the literature, oridonin can markedly inhibit experimental autoimmune neuritis (EAN) by lessening local inflammatory reaction and increasing the proportion of immune regulating macrophages in the peripheral nerves possibly by the pathway of Notch, which indicates that it can be developed as a potential therapeutic agent for human Guillain-Barre syndrome (GBS) and neuropathies (Xu L. et al., 2019). Moreover, the employment of oridonin enables to relieve carrageenan-induced pleurisy through activating the KEAP-1/Nrf2 pathway and suppressing the TXNIP/NLRP3 and NF-*κ*B pathway in the model of BALB/c mice. These specific manifestations includes reducing lung injury scores, releasing of cytokines, neutrophil infiltration, exudating volume and the exudate protein concentration, decreasing the levels of oxidative stress markers (Yang et al., 2020). Recently, researcher relies on the fact that oridonin itself can act as a protective agent against LPS-induced inflammatory response, which the specific mechanisms involve in ROS accumulation, JNK activation, nuclear translocation of NF-*κ*B (Huang et al., 2020). Oridonin also inhibits autophagy and survival in rheumatoid arthritis fibroblast-like synoviocytes (He et al., 2020). In addition, oridonin can also resist a series of inflammatory reactions including LPS-induced inflammation in human gingival fibroblasts (Yu et al., 2019), IL-1β-induced inflammation in human osteoarthritis chondrocytes (Jia et al., 2019) and LPS-induced endometritis (Zhou et al., 2019). These findings indicate that oridonin may be served as a potential therapeutic agent for a variety of inflammatory related diseases. A great deal of immune cells including T cells plays an important role in the process of inflammation. In recent years, studies on anti-inflammatory effect of oridonin based on immune response have gradually increased. Research showed that it alleviated the colitis induced by trinitrobenzene sulfonic acid as represented by a decrease in colonic interferon-/inteleukin-17 secretion and a consumption in splenic Th1/Th17 cells and effector memory CD4(+) T cells (Wang et al., 2015). In addition, oridonin inhibited inflammatory graft rejection by depleting a great number of T cells in spleen and peripheral blood (Guo et al., 2013).

### Anticancer Activity

The efficacy of mainstay cancer therapies such as cytotoxics and radiation, has reached a plateau in the treatment of multiple cancers. In this regard, there is an urgent sense that ameliorations must now come from fresh approaches. In recent years, continuous attention is also shifting to the development of natural anti-tumor agents. Oridonin has a variety of documented anti-cancer activities such as its ability to against gastric cancer (He et al., 2017), oral cancer (Yang Y.-C. et al., 2017), nasopharyngeal carcinoma (Liu et al., 2021), esophageal cancer (Jiang et al., 2019), ovarian cancer (Dong et al., 2018), leukemia (Li and Ma, 2019; Zhang D. et al., 2019), and myeloma (Wu et al., 2020), etc. Its main mechanism involves in inhibiting proliferation (Hao et al., 2016), inducing apoptosis (Gu et al., 2015; Clayton et al., 2016; Qing et al., 2016; Xu et al., 2016) and autophagy (Tiwari et al., 2015; Yao et al., 2017), suppressing migration and invasion (Li Y.-C. et al., 2016), reversing drug resistance (Kadioglu et al., 2018)] and so on.

**FIGURE 2:**
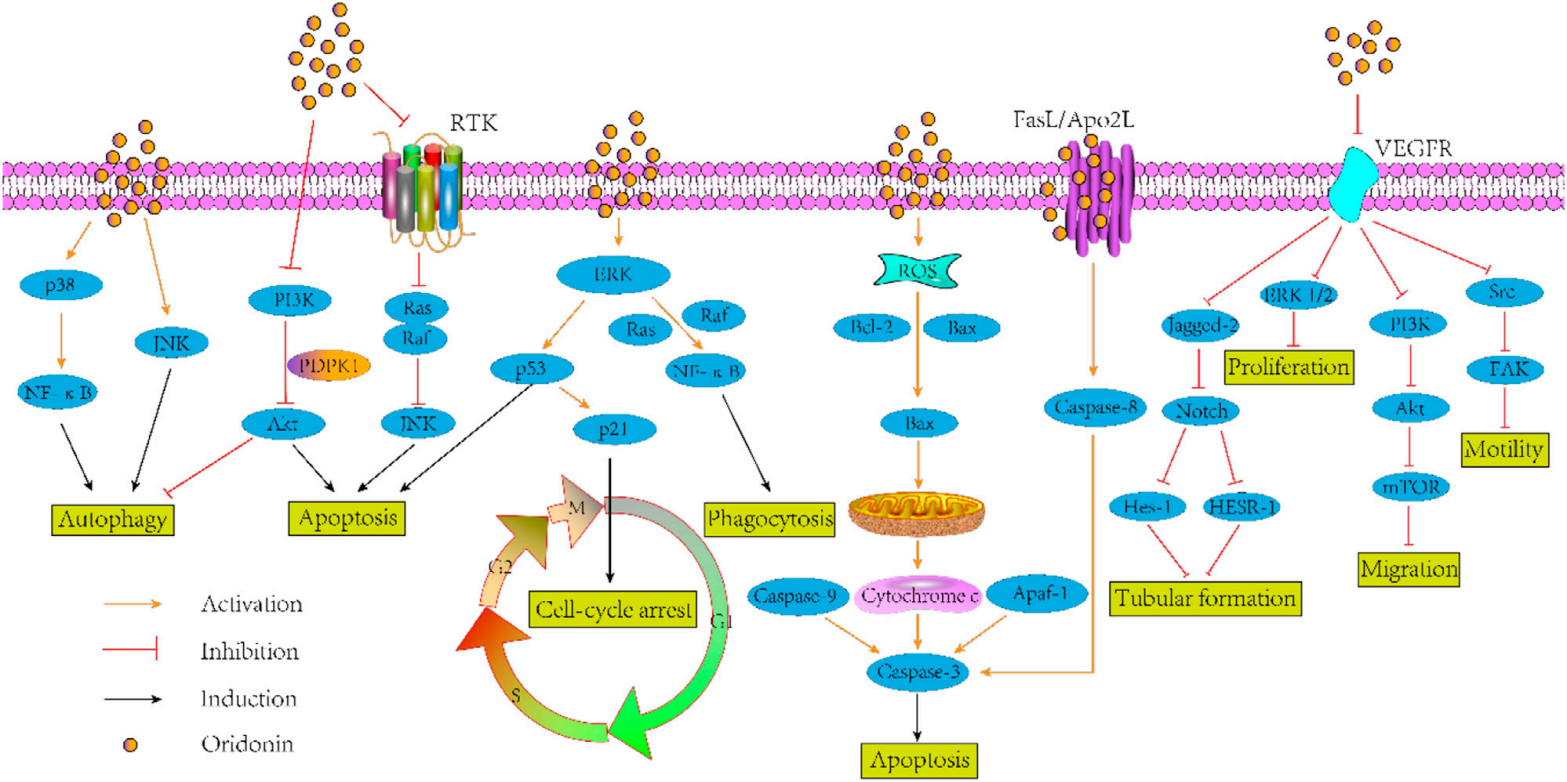
The antitumor mechanism of oridonin.

As documented in literature, utilization of oridonin increased the level of E-cadherin and ALP, reduced the vimentin, phospho-FAK levels, snail, slug, and LDH in human small cell lung cancer cell line H1688 with concentration of 2.5, 5, 10, 20, and 40 µM for 24 and 48 h *in vitro*. Of course, the author also confirmed the anti-lung cancer effect of oridonin in the model of BALB/c nude mice (Xu et al., 2020). Another study on the anti-lung cancer of oridonin proved that, oridonin sensitized cisplatin-induced apoptosis *via* AMPK/Akt/mTOR-dependent autophagosome accumulation in A549 Cells (Yang et al., 2019a). Moreover, it augmented the radiosensitivity of lung cancer cells by up-regulating Bax and down-regulating Bcl-2 (Li C. et al., 2018), underpinned radiation-induced cell death by accelerating DNA damage in non-small cell lung cells (Park et al., 2018) and promoted G_2_/M arrest in A549 cells by facilitating ATM (Zheng et al., 2017). In the aspect of anti-breast cancer, oridonin could synergistically enhance the anti-tumor effect of doxorubicin on aggressive breast cancer by promoting apoptosis and anti-angiogenesis (Li et al., 2019). Besides, this compound could inhibit angiogenesis and EMT related to VEGF-A (Li C. Y. et al., 2018), block Notch signaling pathway to inhibit the growth and metastasis of breast cancer (Xia et al., 2017), and induce autophagy to promote apoptosis (Li and Yang, 2015). In addition to its above anti-tumor effects, there is growing evidences that oridonin exhibits other anti-tumor activities such as colorectal cancer (Bu H. et al., 2019), pancreatic cancer (Liu D. et al., 2020), gallbladder cancer (Chen, et al., 2019), prostate cancer (Lu et al., 2017) and so on. Given that pathway defects have been recognized by most chemotherapies, oridonin may be a logical botanical for future researches of tumor adjuvant therapy. Figure 2 gives the antitumor mechanism of oridonin.

### Hepatorenal Protective Activity

With the deepening of the research, the hepatorenal protective activity of oridonin has been gradually recognized. In a report on the research of LPS/D-galactosamine-induced acute liver injury in mice, oridonin was used as a compound known to be effective at improving the survival rate, alleviating histopathological abnormalities, and suppressing plasma aminotransferases, which the mechanisms may involve in the suppression of pro-apoptotic cytokine TNF-α and JNK-associated pro-apoptotic signaling (Deng et al., 2017). Oridonin also ameliorated carbon tetrachloride-induced liver fibrosis in mice through inhibiting the NLRP3 inflammasome (Liu D.-L. et al., 2020). Mouse immortalized stellate cell line JS1 treated with oridonin at the concentration of 5, 10, and 15 µM showed that it significantly impede posttranslational modifications of IRAK4 in the TLR4 signaling pathway (Shi et al., 2019). In addition, the inhibition of LPS induced apoptosis promoting cytokines IL-1 β, IL-6, and MCP-1, as well as ICAM-1 and VCAM-1 observed in LX-2 cells also appear to be able to validate the protective effect of oridonin on liver (Cummins et al., 2018). In terms of kidney protection, oridonin alleviated IRI-induced kidney injury by suppressing inflammatory response of macrophages through AKT-related pathways (Yan et al., 2020). Furthermore, oridonin at the concentrations of 2.5, 5, 10, and 20 µM managed to alleviate albuminuria, improve renal function and attenuate renal histopathological injury, hinder inflammatory cytokine production, down-regulate TLR4 expression and inhibit NF-*κ*B and p38-MAPK activation, with the effects augmented as the dose increased (Li J. et al., 2018). These studies may provide a new recognition of natural medicine for the treatment of liver and kidney diseases.

### Cardioprotective Activity

Diseases associated with cardiovascular diseases are an increasing problem in most parts of the world and, as with many other problems of today, are becoming more and more urgent for people all over the world. Therefore, a reasonable and effective strategy and approach is now essential to fight against this malady. As reported by researches in recent years, oridonin exhibited beneficial influences on cardiovascular disease. In a myocardial ischemia-reperfusion injury mouse models, down-regulation of oxidative stress and NLRP3 inflammation has been shown to mitigative effect of oridonin to myocardial ischemia reperfusion injury (Lu et al., 2020). Similar results have been verified by researchers from the perspective of metabonomics (Zhang J. et al., 2019). Oxidative stress, which has a critical link with the development of cardiac hypertrophy and heart failure, can reportedly be inhibited by oridonin *via* mitigating pressure overload-induced cardiac hypertrophy and fibrosis, preserving heart function, enhancing myocardial autophagy in pressure-overloaded hearts and angiotensin II-stimulated cardiomyocytes (Xu M. et al., 2019). In the respect of inhibition for vascular inflammation, oridonin could reduce the endothelial-leukocyte adhesion and leukocyte transmigration, inhibit the expression of TNF-α-induced endothelial adhesion molecules, suppress the penetration of the leukocyte, suppress the TNF-α-activated MAPK and Nuclear factor kappa B (NF-*κ*B) activation, as described in the literature (Huang et al., 2018).

### Lung Protective Activity

In recent years, oridonin, isolated from the plants of the genus rubescens, has shown great potential in lung protection due to its antioxidant and anti-inflammatory effects. Oxidative stress and the resulting inflammation are significant pathological processes in acute lung injury (ALI). According to the literature, oridonin can exert protective effects on LPS-induced ALI through Nrf2-independent anti-inflammatory and Nrf2-dependent anti-oxidative activities (Yang et al., 2019b). It also protects against chemical induced pulmonary fibrosis. Research shows that it could markedly suppress the mRNA and protein expression of *α*-SMA and COL1A1 in TGF-b1-induced MRC-5 cells as well as undermine pathological changes, such as alveolar space collapse, emphysema, and infiltration of inflammatory cells induced by BLM (Fu et al., 2018). Immune regulation disorder and persistent inflammatory injury are important mechanisms of ventilator-induced lung injury (VILI). As research has shown, oridonin can reduce VILI by blocking the interaction between NEK7 and NLRP3 and halting the activation of NLRP3 inflammatory bodies (Liu H. et al., 2020). In addition, post-exposure treatment with oridonin was able to ameliorate lung pathology, attenuate lung edema, abate MDA and TNF-α, and elevate GSH and IL-10 in the lung, which indicate that it can defend the lung against hyperoxia-induced injury in the model of mice (Liu et al., 2017).

### Neuroprotective Activity

Oridonin produced a conspicuous effect of neuroprotective in PC12 and N2a cells by rescuing IR, reducing the autophagosome formation and synaptic loss and ameliorating cognitive dysfunction, halting IR-induced synaptic deficits (Wen et al., 2020). In the A*β*_1–42_-induced mouse model of Alzheimer’s disease (AD), oridonin sharply rescues synaptic loss induced by A*β*_1–42_, lessens the alterations in dendritic structure and spine density, augment PSD-95 and promotes mitochondrial activity (Wang J. et al., 2016). The neuropathological characteristics of AD are amyloid aggregation, tau phosphorylation, and neuroinflammation. A study indicates that different routes of administration of oridonin severely attenuated-amyloid deposition, plaque-associated APP expression and microglial activation, which suggest that this natural terpenoid might be considered a prospective therapeutic agent for human neurodegenerative diseases such as AD (Zhang et al., 2013). Furthermore, available data suggest the potentiality of oridonin to attenuate A*β*_1–42_-induced neuroinflammation and inhibit NF-*κ*B pathway (Wang et al., 2014).

### Other Pharmacological Activities

Several lines of evidence suggest oridonin exerts its potential role of amelioration lupus-like symptoms through suppressing BAFF expression, improving serological and clinical manifestations of SLE, lessening proteinuria levels, diminishing production of specific auto-antibodies (Zhou et al., 2013). Besides, oridonin exerted its protective effects against hydrogen peroxide-induced damage by altering the profiles of mRNA in human dermal fibroblasts (Lee et al., 2013). In the treatment of respiratory diseases, oridonin could lessen protein quantification in bronchoalveolar lavage fluid and the lung W/D ratio, mitigate inflammation and suppress the injuries, as well as abate the TNF-α, IL-6 (Jiang et al., 2017). Oridonin could also decrease the OVA-induced airway hyper-responsiveness and eosinophil number, and suppress the eosinophilia and mucus production, which confirms its great prospect in the treatment of asthma (Wang S. et al., 2016). In addition, oridonin could effectively ameliorate inflammation-induced bone loss in the model of mice by inhibiting DC-STAMP expression (Zou et al., 2020), halt the growth of methicillin-resistant *Staphylococcus aureus* (MRSA) (Yuan et al., 2019), mitigate visceral hyperalgesia in a rat model of postinflammatory irritable bowel syndrome (Zang et al., 2016), and augment gamma-globin expression in erythroid precursors from patients (Guo et al., 2020).

Due to the extensive biological effects of oridonin, its application in aquaculture has been gradually discovered in recent years. As reported in the literature, oridonin could improve the antioxidant capacity of arbor acres broilers liver, as evidenced by the decrease in MDA and the increase in total SOD activities and mRNA expression levels of the liver antioxidant genes (Zheng, et al., 2016). Adding oridonin to the diet of arbor acres broilers could significantly improve the immune response induced by *Salmonella* and protect the intestinal health (Wu et al., 2018a), increase the relative weights of spleen and bursa, number of proliferation peripheral blood T and B lymphocytes, the phagocytic rate of neutrophils, as well as the IL-2, IL-4, and TNF-α (Wu et al., 2018a). In addition, oridonin could also interfere with the effects of *Salmonella pullorum* on immune cells and Th1/Th2 balance of spleen in arbor acres broilers (Wu et al., 2018b). As discussed above, oridonin is a natural active compound with therapeutic potential for dozens of diseases. Additional details on the pharmacological activities of oridonin were depicted as in Table 1.

**TABLE 1:** Pharmacology of oridonin.

Pharmacological effects	Detail	Cell lines/Model	Dose	Application	Ref
Anti-inflammatory activity	Reduce lung injury scores, cytokines, neutrophil infiltration, and exudate volume and exudate protein concentration, decrease oxidative stress markers	BALB/c mice	5–20 mg/kg	*In vivo*	Yang et al. (2020)
	Prevent ROS accumulation, attenuate RAW 264.7 cell chemotaxis toward LPS-treated HK-2 cells	HK-2 cells	30 μg/ml	*In vitro*	Huang et al. (2020)
		RAW 264.7	30 μg/ml	*In vitro*	
	Suppress proliferation, increase apoptosis and Bax and cleaved caspase-3 but decrease the IL-1b, inhibit ATG5 and Beclin1	RA-FLSs	2–12 μg/ml	*In vitro*	He et al. (2020)
	Inhibit inflammatory mediators PGE2, NO, IL-6, and IL-8, reduce phosphorylation of NF-*κ*B p65 and I*κ*B*α*, up-regulate PPAR-*γ*	Human gingival fibroblasts	10–30 μg/ml	*In vitro*	Yu et al. (2019)
	Suppress IL-1β-induced MMP1, MMP3, and MMP13, attenuate IL-1β-induced NO and PGE2, as well as iNOS and COX-2, reduce IL-1β-induced NF-*κ*B activation	Human chondrocytes	10–30 μg/ml	*In vitro*	Jia et al. (2019)
	Alleviate LPS-induced endometritis and reduce the activity of myeloperoxidase, decrease TNF-α, IL-1β, and IL-6, inhibit LPS-induced TLR4/NF-*κ*B signaling pathway activation	BALB/c mice	40 mg/kg	*In vivo*	Zhou et al. (2019)
		mEECs	10–100 μg/ml	*In vitro*	
	Relieve hypoxia-evoked apoptosis and autophagy *via* modulating microRNA-214	H9c2 cells	1–20 µM	*In vitro*	Gong et al. (2019)
	Inhibit pro-inflammatory cytokines, such as IL-1β, IL-6, and TNF-α, through the TLR4/MyD88/NF-*κ*B axis	BALB/c mice	10–40 mg/kg	*In vivo*	Zhao G. et al. (2017)
		RAW264.7 cells	5–40 μg/ml	*In vitro*	
	Inhibits IL-1β-induced proliferation and phosphorylation of MAPK, promote apoptosis and increase intracellular ROS.	Primary human FLSs	5–40 µM	*In vitro*	Shang et al. (2016)
	Protect HaCaT keratinocytes against hydrogen peroxide-induced oxidative stress by altering microRNA expression	HaCaT keratinocytes	1–20 µM	*In vitro*	Bae et al. (2014)
Anticancer activity	Increase the level of E-cadherin and ALP, reduce the vimentin, phospho-FAK levels, snail, slug, and LDH, and inhibit tumor growth in mouse model	H1688 cells	2.5–40 µM	*In vitro*	Xu et al. (2020)
		BEAS-2B cells	2.5–40 µM	*In vitro*	
		HBE cells	2.5–40 µM	*In vitro*	
		BALB/c mice	5–10 mg/kg	*In vivo*	
	Enhance cisplatin sensitivity *via* pro-apoptotic activity mediated by AMPK/Akt/mTOR-dependent autophagosome activation	A549 cells	5–30 µM	*In vitro*	Yang et al. (2019a)
		B2b cells	5–30 µM	*In vitro*	
		C57BL/6 WT mice	20 mg/kg	*In vivo*	
	Inhibit the proliferation in a time- and dose-dependent manner, enhance the radiosensitivity of SPC-A-1 cells, increase Bax and decrease the Bcl-2	HCC827 cells	10–80 µM	*In vitro*	Li C. et al. (2018)
		SPC-A-1 cells	10–80 µM	*In vitro*	
	Enhance radiation-induced inhibition of cell growth and clonogenic survival, facilitate radiation-induced ROS production and DNA damage and enhance apoptotic cell death	NCI-H460 cells	1–5 µM	*In vitro*	Park et al. (2018)
		BALB/c mice	15 mg/kg	*In vivo*	
	Inhibit proliferation by inducing cycle arrest at G2/M through ATM-p53-CHK2 pathway	A549 cells	16–64 µM	*In vitro*	Zheng et al. (2017)
	Increase the intracellular accumulation of Dox, decrease proliferation, migration, invasion and tube formation, reverse Dox-induced cardiotoxicity	MDA-MB-231 cells	0.6–20 µM	*In vitro*	Li et al. (2019)
		HUVECs cells	2.5 µM	*In vitro*	
		BALB/c nude mice	16 mg/kg	*In vivo*	
	Suppress migration, invasion and adhesion, inhibit tube formation and EMT, decrease N-cadherin, Vimentin and Snail, HIF-1α, VEGF-A and VEGF receptor-2 protein expression	BALB/c mice	2.5–10 mg/kg	*In vivo*	Li C. Y. et al. (2018)
		MDA-MB-231 cells	2–64 µM	*In vitro*	
		MCF-10A cells	2–64 µM	*In vitro*	
	Induce cells apoptosis, inhibit cancer cell migration and invasion, and decrease the expression of Notch 1–4 protein	4T1 cells	0.1–10 mM	*In vitro*	Xia et al. (2017)
		BALB/C athymic mice	10–20 mg/kg	*In vivo*	
	Inhibit proliferation, induce apoptosis, up-regulate Bax and down-regulate Bcl-2, increase cleaved caspase-9 and LC3-II.	MDA-MB-436 cells	10–80 µM	*In vitro*	Li et al. (2015)
		MDA-MB-231 cells	10–80 µM	*In vitro*	
	Inhibit proliferation and induce apoptosis, reduce *β*-catenin, increase GSK3*β* and decrease phosphorylation of GSK3*β*, suppress tumor growth	COLO205 cells	5–25 µM	*In vitro*	Bu H. et al. (2019)
		BALB/c nude mice	10–20 mg/kg	*In vivo*	
	Inhibit proliferation, induce cellular morphology changes and Bax translocation from cytosolic to mitochondrial compartments, and suppress tumor growth	BxPC-3 cells	5–80 µM	*In vitro*	Liu D. et al. (2020)
		PANC-1 cells	5–80 µM	*In vitro*	
		BALB/c nude mice	40 mg/kg	*In vivo*	
	Suppress proliferation, induce apoptosis and cell cycle arrest at the G_0_/G_1_ phase, down-regulate HIF-1α/MMP-9	GBC-SD cells	5–20 µM	*In vitro*	Chen et al. (2019)
		BALB/c nude mice	15 mg/kg	*In vivo*	
	Inhibit proliferation and induce G2/M cell cycle arrest and apoptosis, up-regulate p53, p21, proteolytic cleaved forms of caspase-3, caspase-9, decrease B-cell lymphoma 2	PC3 cells	20–60 µM	*In vitro*	Lu et al. (2017)
		DU145 cells	20–60 µM	*In vitro*	
	Inhibit proliferation, invasion, and migration, down-regulate phosphorylation of EGFR, ERK, Akt, expression of MMP-12 and CIP2A, inhibit tumor growth *in vivo*	A549 cells	40–90 µM	*In vitro*	Xiao et al. (2016)
		NCI-H1975 cells	5–30 µM	*In vitro*	
		Nude mice	30 mg/kg	*In vivo*	
	Elevate cisplatin-caused reduction of cell viabilities and enhance cell apoptosis, inhibit autophagy	A2780CP cells	5–40 µM	*In vitro*	Zhao and Xia, (2019)
		SKOV3 cells	5–30 µM	*In vitro*	
		DDP cells	5–30 µM	*In vitro*	
	Suppress the proliferation and block the cell cycle in G1/S phage and induce apoptosis	SKOV3 cells	5–50 µM	*In vitro*	Wang et al. (2019)
		A2780 cells	5–50 µM	*In vitro*	
		HL-7702 cells	5–50 µM	*In vitro*	
	1Reverse cisplatin resistance, induce apoptosis and promote cell-cycle arrest, down-regulate Bcl-2 and up-regulate Bax protein, decrease MMP-2 and MMP-9	A2780 cells	10–160 µM	*In vitro*	Ma S. et al. (2016)
		SKOV3 cells	10–160 µM	*In vitro*	
	Induce ROS accumulation and cell apoptosis *via* the c-Jun N-terminal kinase (JNK)/c-Jun pathway	DLD1 cells	10–90 µM	*In vitro*	Zhang D. et al. (2019)
		RKO cells	10–90 µM	*In vitro*	
		LS174T cells	10–90 µM	*In vitro*	
		SW480 cells	10–90 µM	*In vitro*	
		SW48 cells	10–90 µM	*In vitro*	
		HCT116 cells	10–90 µM	*In vitro*	
		HCT-15 cells	10–90 µM	*In vitro*	
	Inhibit proliferation, reduce Smad2, Smad3, Smad4, PAI-1 and the phosphorylation of Smad2 and Smad3 induced by TGF-β1 *in vitro* and suppress tumor growth *in vivo*	LOVO cells	2–16 μg/ml	*In vitro*	Bu H.-Q. et al. (2019)
		SW480 cells	2–16 μg/ml	*In vitro*	
		HT29 cells	2–16 μg/ml	*In vitro*	
		BALB/c nude mice	2.5,5,7.5 mg/kg	*In vivo*	
	Inhibit proliferation and induce apoptosis, increase total and phosphorylated levels of p53, increase the expression of BMP7, reduce the growth rate of tumors in mice	HCT116 cells	5–25 µM	*In vitro*	Liu R.-X. et al. (2018)
		SW620 cells	5–25 µM	*In vitro*	
		SW480 cells	5–25 µM	*In vitro*	
		LoVo cells	5–25 µM	*In vitro*	
		FHC cells	5–25 µM	*In vitro*	
		Athymic nude mice	50–100 mg/kg	*In vivo*	
	Inhibit the proliferation and induce the apoptosis, up-regulate BMP7 and increase the level of phosphorylated p38 MAPK.	HCT116 cells	5–25 µM	*In vitro*	Ren et al. (2016)
	Inhibit proliferation, induce cell cycle arrest and apoptosis and inhibit tumor growth, increase the total protein level of PTEN and reduce the phosphorylation of PTEN.	HCT116 cells	5–80 µM	*In vitro*	Wu et al. (2016)
		Athymic nude mice	50–100 mg/kg	*In vivo*	
	Inhibit proliferation, induce apoptosis, arrest cell cycle, prevent migration, regulate EMT-related protein expression, and inhibit cell tumorigenicity and EMT in nude mice	BxPC-3 cells	20–160 µM	*In vitro*	Lou et al. (2019)
		PANC-1 cells	20–160 µM	*In vitro*	
		BALB/C nude mice	10 mg/kg	*In vivo*	
	Lead to a dose-dependent reduction of clonogenic survival and an increase in γH2AX, observe additive effects and a prolonged G2/M-arrest	AsPC-1 cells	0.5–2.5 μg/ml	*In vitro*	Liermann et al. (2017)
		BxPC-3 cells	0.5–2.5 μg/ml	*In vitro*	
		MIA PaCa-2 cells	0.5–2.5 μg/ml	*In vitro*	
	Inhibit proliferation, downregulate miR-200b-3p, inhibit migration, EMT and ZEB1, N-cadherin and fibronectin. *In vivo*, inhibit migration in the nude mouse model	BxPC-3 cells	20–160 µM	*In vitro*	Gui et al. (2017)
		PANC-1 cells	20–160 µM	*In vitro*	
		BALB/C nude mice	10 mg/kg	*In vivo*	
	Overcome PANC-1/Gem cells gemcitabine reistance by regulating GST pi and LRP1/ERK/JNK signaling	PANC-1 cells	10–160 µM	*In vitro*	Wang and Zhu (2019)
		PANC-1/Gem cells	10–160 µM	*In vitro*	
	Inhibit proliferation and potentiate gemcitabine-induced apoptosis, up-regulate the pro-apoptotic genes Bax, cytochrome c (cyt c), and caspase-3 and-9	PANC-1 cells	20–100 µM	*In vitro*	Liu et al. (2014)
	105 mRNAs were differentially expressed	BxPC-3 cells	87.8 µM	*In vitro*	Gui et al. (2015)
	Cause a perturbation in mitochondrial redox status	HepG2 cells	5–60 µM	*In vitro*	Liu X. et al. (2018)
	Increase the anticancer effects	L02 cells	4–40 µM	*In vitro*	Sun Y. et al. (2018)
		HepG2 cells	4–40 µM	*In vitro*	
	Increase the inhibitory effect on tumor cells and induce apoptosis	SMMC-7721 cells	4–40 µM	*In vitro*	Xu et al. (2017)
	Induce apoptosis and regulate expression and activity of apoptosis-related proteins, down-regulate nuclear translocation of p50 and p65, decrease the transcription activity of all NF-kappa B subunits	HepG2 cells	0.5–50 μg/ml	*In vitro*	Dong et al. (2016)
	Induce tumor cell necroptosis by reducing GSH and enhancing ROS formation, enhance cytotoxic effect of 5-FU.	786-O cells	10–40 µM	*In vitro*	Zheng et al. (2018)
		Nude mice	20 mg/kg	*In vivo*	
	Suppress cell viability and inhibit cell proliferation by inducing G2/M arrest, induce caspase-dependent apoptosis	HGC-27 cells	2.5–15 µM	*In vitro*	Ren et al. (2020)
	Inhibit proliferation, migration, and survivability, enhance apoptosis and the anti-tumor effect of cisplatin, up-regulate mRNA and protein expression of p53	SNU-216 cells	10–80 µM	*In vitro*	Bi et al. (2018)
	Inhibit proliferation, induce apoptosis, down-regulate Bcl-2 and up-regulate Bax, induce the release of cytochrome c	SGC-7901 cell	2–8 µM	*In vitro*	Gao et al. (2016)
	Inhibit P300, GCN5, Tip60, and pCAF, inhibit proliferation and down-regulate p53, induce apoptosis, increase activated caspase-3 and caspase-9, decrease the mitochondrial membrane potential	AGS cells	1–100 µM	*In vitro*	Shi et al. (2016)
	Suppress proliferation and soft agar colony formation, induce ROS-dependent apoptosis by mitochondrial-dependent pathway	HN22 cells	5–10 µM	*In vitro*	Oh et al. (2018)
	Enhance the mitochondrial apoptosis through NF-*κ*B, induce ROS production	HEp-2 cells	12–36 µM	*In vitro*	Kang et al. (2020)
		Tu212 cells	12–36 µM	*In vitro*	
	Result in apoptosis and induce autophagy, increase the binding NF-*κ*B family member p65 with the promotor of BECN 1	HEp-2 cells	24 µM	*In vitro*	Cao et al. (2019)
		Tu212 cells	24 µM	*In vitro*	
	Target caspase-9 to alter ROS production and autophagy to promote cell apoptosis	HEp-2 cells	36 µM	*In vitro*	Kang et al. (2015)
	Induce ROS-mediated cell apoptosis	KYSE-150 cells	10–50 µM	*In vitro*	Pi et al. (2015)
	Induce apoptosis, increase the t-Bid as a downstream target of MCL-1 and decrease mitochondrial membrane potential	MC-3 cells	7.5–30 µM	*In vitro*	Han et al. (2020)
		YD-15 cells	6.25–25 µM	*In vitro*	
	Exhibit anti-RUNX1-ETO activity, and ERK2 kinase inhibitors, cause decrease of phosphorylated ERK1/2	Kasumi-1 cells	1–5 µM	*In vitro*	Spirin et al. (2017)
		U937 cells	1–5 µM	*In vitro*	
		Jurkat cells	1–5 µM	*In vitro*	
	Inhibit EMT, prevent TGF-β1-induced EMT by inhibiting Smad2/3 pathway and osteosarcoma metastasis to lung in the metastatic model	MG-63 cells	0.5–2 µM	*In vitro*	Sun Z. et al. (2018)
		143B cells	0.5–2 µM	*In vitro*	
		U-2OS cells	0.5–2 µM	*In vitro*	
		Nude mice	15 mg/kg	*In vivo*	
	Inhibit expression of protein that related to cell proliferation	LP-1 cells	5–50 µM	*In vivo*	Zhao J. et al. (2017)
	Exert its anticancer activity partially by targeting the Mdm2-p53 axis in NB cells	SH-SY5Y cells	2–20 µM	*In vitro*	Zhu et al. (2019)
		SK-N-SH cells	2–20 µM	*In vitro*	
		SK-N-MC cells	2–20 µM	*In vitro*	
	Suppress proliferation, induce apoptosis, downregulates the Wnt/*β*-catenin signaling pathway	Neurocytoma cells	5–25 µM	*In vitro*	Liang et al. (2018)
	Inhibit migration, invasion, adhesion and TGF-β1-induced EMT by inhibiting the activity of PI3K/Akt/GSK-3β signaling pathway	A375 cells	5–40 µM	*In vitro*	Li J. et al. (2018)
		B16-F10 cells	5–40 µM	*In vitro*	
	Down-regulate VEGFR2-mediated FAK/MMPs, mTOR/PI3K/Akt and ERK/p38 signaling pathways	HUVECs	2.5–20 µM	*In vitro*	Jiang et al. (2020)
	Inhibit proliferation, migration, invasion, and tube formation and induce apoptosis, decrease VEGFA, VEGFR2, and VEGFR3 expressions, while increase the TP53	HUVECs	39–312 μg/ml	*In vitro*	Tian et al. (2017)
		Zebrafish	50–200 μg/ml	*In vivo*	
Hepatorenal protective activity	Attenuate liver injury and reduce ALT levels, Sirius Red staining and the *α*-SMA, downregulate NLRP3, caspase-1, and IL-1β and decrease the expression of F4/80	C57BL/6J mice	5 mg/kg	*In vivo*	Liu D.-L. et al. (2020)
		LX-2 cells	1.25 µM	*In vitro*	
	Impede posttranslational modifications of IRAK4 in the TLR4 signaling pathway	JS1 cells	5–15 µM	*In vitro*	Shi et al. (2019)
	Inhibit proinfammatory cytokines IL1-beta, IL-6, MCP-1, cell adhesion molecules ICAM-1 and VCAM-1, block LPS-induced NF-*κ*B p65 nuclear translocation and DNA binding activity	LX-2 cells	2.5–7.5 µM	*In vitro*	Cummins et al. (2018)
	Alleviate albuminuria, improve renal function and attenuate histopathological injury, decrease inflammatory cytokine, down-regulate TLR4 and inhibit NF-*κ*B and p38-MAPK activation	SD rats	10 mg/kg	*In vivo*	Li S. et al. (2018)
		Rat mesangial cell	2.5–20 µM	*In vitro*	
	Inhibit LX-2 and HSC-T6 proliferation, induce apoptosis and S phase arrest, decrease α-SMA and ECM protein type I collagen and fibronectin, block TGF-β1-induced Smad2/3 phosphorylation and type I Collagen expression	LX-2 cells	2.5–30 µM	*In vitro*	Bohanon et al. (2014)
		HSC-T6 cells	2.5–30 µM	*In vitro*	
Cardioprotective activity	Alleviate myocardial injury induced *via* inhibiting the oxidative stress and NLRP3 inflammasome pathway	C57BL/6 mice	10 mg/kg	*In vivo*	Lu et al. (2020)
	Decrease infarct size and reverse abnormal elevated myocardial zymogram, regulate glycolysis, branched chain amino acid, kynurenine, arginine, glutamine and bile acid metabolism	C57BL/6 mice	10 mg/kg	*In vivo*	Zhang W. et al. (2019)
	Mitigate pressure overload-induced cardiac hypertrophy and fibrosis, preserve heart function, and enhance myocardial autophagy	NRCMs	5–50 µM	*In vitro*	Xu L. et al. (2019)
		C57BL/6 mice	40 mg/kg	*In vivo*	
	Reduce endothelial-leukocyte adhesion and leukocyte transmigration, inhibit TNF-α-induced endothelial adhesion molecules, suppress penetration of the leukocyte, and suppress TNF-α-activated MAPK and NF-κB activation	HUVECs	0.5–1,5 µM	*In vitro*	Huang et al. (2018)
		C57BL/6J mice	35 mg/kg	*In vivo*	
Lung protective activity	Increase Nrf2 and HO-1, GCLM, inhibit LPS-induced activation of the pro-inflammatory pathways NLRP3 inflammasome and NF-*κ*B pathways	C57BL/6 mice	20–40 mg/kg	*In vivo*	Yang et al. (2019b)
		RAW 264.7 cells	2.5–10 µM	*In vitro*	
	Inhibit myofibroblast differentiation and bleomycin-induced pulmonary fibrosis by regulating TGF-beta/smad pathway	Kunming mice	10–20 mg/kg	*In vivo*	Fu et al. (2018)
		MRC-5 cells	2.5–20 µM	*In vitro*	
Neuroprotective activity	Rescue IR, reduce the autophagosome formation and synaptic loss and improve cognitive dysfunction, block IR-induced synaptic deficits	SD rats	5 mg/kg	*In vivo*	Wen et al. (2020)
		PC12 cells	0.05–5 µM	*In vitro*	
		N2a cells	0.05–5 µM	*In vitro*	
	Rescue synaptic loss induced by Aβ_1-42_, attenuate alterations in dendritic structure and spine density, increase PSD-95 and synaptophysin and promote mitochondrial activity, activate BDNF/TrkB/CREB signaling pathway	C57BL/6 (B6) mice	10–50 mg/kg	*In vivo*	Wang J. et al. (2016)
	Attenuate b-amyloid deposition, plaque-associated APP expression and microglial activation, ameliorate deficits in nesting and inflammatory reaction of macrophage and microglial cell lines	APP/PS1-21 mice	20 mg/kg	*In vivo*	Zhang et al. (2013)
		RAW 264.7 cells	1 μg/ml	*In vitro*	
		N9 cells	1 μg/ml	*In vitro*	
	Inhibit pro-inflammatory factors in hippocampus, ameliorate microglia and astrocytes activation.	Ab1–42 induced AD mice	10 mg/kg	*In vivo*	Wang et al. (2014)
Other pharmacological activities	Inhibit BAFF expression, ameliorate serological and clinical manifestations of SLE, reduce proteinuria levels, diminish production of specific auto-antibodies, and attenuate renal damage	MRL^lpr/lpr^ mice	4.5–18 mg/kg	*In vivo*	Zhou et al. (2013)
		RAW264.7 cells	3–24 μg/ml	*In vitro*	
	Against hydrogen peroxide-induced damage by altering mRNA expression	NHDF cells	1–20 µM	*In vitro*	Lee et al. (2013)
	Reduce protein quantification in bronchoalveolar lavage fluid and lung W/D ratio, relieve inflammation and reduce the injuries, decrease the TNF-alpha, IL-6	C57BL/6 mice	0.5–50 mg/kg	*In vivo*	Jiang et al. (2017)
	Decrease the OVA-induced airway hyper-responsiveness, eosinophil number and total inflammatory cell, inhibit the eosinophilia and mucus production	BALB/c mice	10, 20 mg/kg	*In vivo*	Wang S. et al. (2016)
	Inhibit mRNA and protein of DC-STAMP, and suppress the following activation of NFATc1 during osteoclastogenesis	RAW264.7 cells	0.39–25 µM	*In vitro*	Zou et al. (2020)
		C57BL/6 mice	2, 10 mg/kg	*In vivo*	
		ICR mice	2, 10 mg/kg	*In vivo*	
	Increase pain threshold pressure, decrease colon EC cell numbers, TPH expression, and serotonin content, increase the spleen index and levels of TNF-α, IFN-γ, IL-4, and IL-13	SD rats	5–20 mg/kg	*In vivo*	Zang et al. (2016)
	Enhance *γ*-globin expression by activating p38 MAPK and CREB1, leading to histone modification in γ-globin gene promoters during the maturation	Human erythroid precursor cells	0.1–1 µM	*In vitro*	Guo et al. (2020)
	Improve antioxidant capacity, as evidenced by the decrease in MDA and the increase in total SOD activities and mRNA expression of the liver antioxidant genes	Arbor Acre broiler chickens	50–100 mg/kg	*In vivo*	Zheng et al. (2016)
	Improve *Salmonella*-induced immune responses and protect intestinal health	Arbor Acre broiler chickens	50–100 mg/kg	*In vivo*	Wu et al. (2018b)
	Increase weights of spleen and bursa, number of proliferation peripheral blood T and B lymphocytes, the phagocytic rate of neutrophils, and the IL-2, IL-4 and TNF-α	Arbor Acre broiler chickens	50–100 mg/kg	*In vivo*	Wu et al. (2018c)

## Pharmacokinetics

**FIGURE 3:**
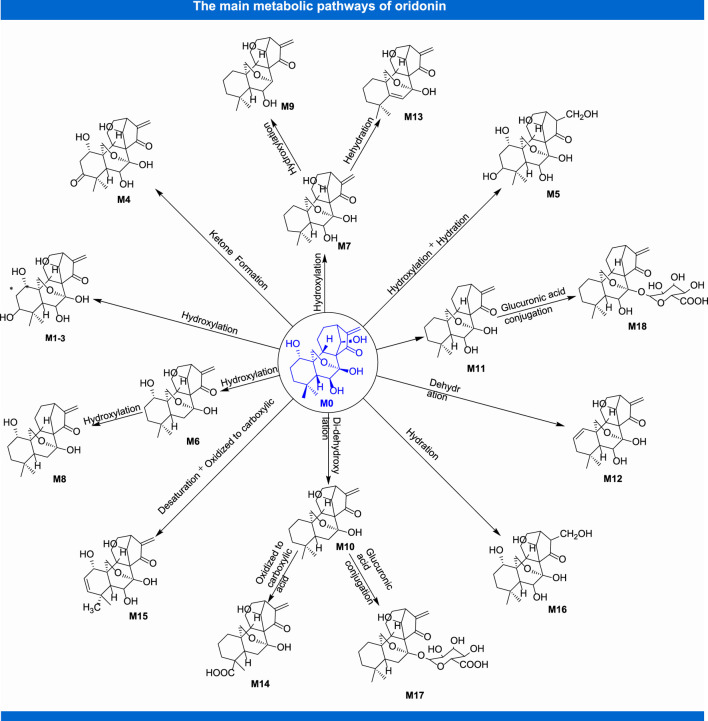
The main metabolic pathways of oridonin.

In the process of innovative agent development, pharmacokinetic research has become a pivotal part of preclinical and clinical research of drugs. It not only plays a supporting role in drug toxicity or clinical research, but also contributes to optimize the screening of candidate agents, which provides a novel approach to study modern pharmacotherapy (Sun et al., 2020a). Up to now, benefited from the continuous emergence of novel analytical techniques, researchers have investigated the pharmacokinetic parameters of oridonin *in vivo* by means of MS-MS (Jin et al., 2010), LC-MS-MS (Du et al., 2010; Jin et al., 2015) and other analytical methods with rats (Jian et al., 2007) and rabbits (Mei et al., 2008), which partially interpreted the kinds of events related to the efficacy and toxicity of relevant herbal preparations in which this constituent is used. Following rat oral administration of *Herba Isodi Rubescentis* extract containing oridonin (1.68 mg/kg), the pharmacokinetic parameters in rat plasma were obtained with the method of LC-MS-MS, revealing AUC0-t at 78.45 ± 33.83 ng/ml/h and AUC_(0-infinity)_ at 79.29 ± 34.26 ng/ml/h, t_1/2_ at 0.19 ± 0.05 h, T_max_ at 0.69 ± 0.13 h, C_max_ at 164.51 ± 58.42 ng/ml (Ma et al., 2013). Determination of oridonin (40 mg/kg) in rat plasma after intragastrical administration with determination of LC-MS-MS suggested that it mainly metabolized in liver, and acquired main pharmacokinetic parameters, such as t_1/2_ at 10.88 ± 4.38 h, T_max_ at 1.00 ± 0.12 h, C_max_ at 146.9 ± 10.17 ng/ml, AUC(0–t) at 1.31 ± 0.29 mg h/L. At the same time, this project also told us that verapamil could substantially alter the pharmacokinetic profile of oridonin in rats, as well as it might exert these effects *via* elevating the absorption of this terpenoid compound by suppressing the activity of P-gp, or through hindering the metabolism of it in rat liver (Liu et al., 2019). Figure 3 shows the main metabolites of oridonin.

A strategy of using ultra-high-performance liquid chromatography-Triple/time-of-flight mass spectrometry (UPLC-Triple-TOF-MS/MS) to identify metabolites and evaluate the *in vitro* metabolic profile of oridonin corroborate that, oridonin is universally metabolized *in vitro*, which the metabolic pathway mainly consists of dehydration, hydroxylation, di-hydroxylation, hydrogenation, decarboxylation, and ketone formation. Meanwhile, 16 metabolites of I- and II-phase were identified (Ma Y. et al., 2016). Another similar study also indicated that 16 phase I and 2 phase II metabolites were detected after oral administration of oridonin in rats, and the main biotransformation pathways of oridonin were reduction, oxidation, dehydroxylation and glucuronic acid coupling (Tian et al., 2015). In addition, the treatment of HepaRG cells with oridonin at concentration of 1, 5, 10, and 20 µM demonstrated that oridonin induced the mRNA and protein expression and enzyme activity of CYP450s, especially on the CYP3A4 and CYP2C9 (Zhang Y. W. et al., 2018). Besides, studies have also shown that oridonin could induce the expression of human CYP3A4 mRNA and protein through pregnane X receptor-mediated (PXR) pathway. Notably, there is no effect on the expression of PXR-nnRNA and protein (Zhang Y.-w. et al., 2014). In the aspect of interaction between oridonin and blood protein, it could bind to human serum albumin (HAS) through hydrogen bonding and van der Waals force, and induce conformational changes of HSA, thus affecting its biological function as carrier protein. The research provides an accurate and full basic data for clarifying the binding mechanism of oridonin with HSA and is beneficial for comprehending its activity on protein function and biological activity *in vivo* during blood transportation process (Li et al., 2015). Other pharmacokinetic studies on oridonin are shown in Table 2.

**TABLE 2:** Pharmacokinetic information of oridonin.

Model	Dose	Administration method	Quantitative method	Detail	Ref
Wistar rats	12.5 mg/kg	Intravenous administration	RP-HPLC method	t_1/2α_ = 0.12 h	Jian et al. (2007)
				t_1/2β_ = 6.06 h	
				CL = 1.56 L/kg/h	
				AUC = 7.96 μg h/ml	
				V_d_ = 1.83 L/kg	
Rabbits	2 mg/kg	Injection administration	HPLC method	t_1/2α_ = 0.11 ± 0.05 h	Mei et al. (2008)
				t_1/2β_ = 2.12 ± 0.87 h	
				CL = 1.44 ± 0.61 h L/kg/h	
				AUC_0–∞_ = 3.53 ± 1.31 μg h/ml	
				V_d_ = 1.72 ± 0.16 h	
				MRT = 2.41 ± 1.07 h	
SD rats	1.68 mg/kg	Intravenous administration	LC–MS-MS method	t_1/2_ = 2.90 ± 0.87 h	Ma et al. (2013)
				CL = 1.08 ± 0.31 h L/kg/h	
				AUC_0–∞_ = 980.74 ± 287.15 ng/ml/h	
				V_d_ = 4.29 ± 0.54 h	
				MRT = 1.79 ± 0.77 h	
SD rats	40 mg/kg	Intragastrical administration	LC-MS/MS method	t_1/2_ = 10.88 ± 4.38 h	Liu et al. (2019)
				CL = 14.69 ± 4.42 h L/kg/h	
				AUC_0–∞_ = 1.31 ± 0.29 mg h/L	
				T_max_ = 1.00 ± 0.12 h	
				MRT = 9.25 ± 1.10 h	
Human liver microsomes	100 µM	Mixed system	UPLC-Triple-TOF-MS/MS and PCA method	The main metabolic pathways of oridonin include dehydration, hydroxylation, dihydroxylation, hydrogenation, decarboxylation and ketogenesis	Ma S. et al. (2016)
Monkey liver microsomes	100 µM				
Rat liver microsomes	100 µM				
Mouse liver microsomes	100 µM				
SD rats	10 mg/kg	Intragastric administration	UPLC-Triple-TOF-MS/MS method	The biotransformation of oridonin mainly includes reduction, oxidation, dehydrogenation and glucuronic acid binding	Tian et al. (2007)
HepaRG cells	1–20 µM	Mixed system	HPLC-MS/MS method	Induce effects on the major member of CYP450s mRNA and protein expression, as well as on the enzyme activity, especially on CYP3A4 and CYP2C9	Zhang et al. (2018b)
HepG2 cells	20 µM	Mixed system	UPLC-MS/MS method	Induce the CYP3A4 reporter luciferase activity, and up-regulate CYP3A4 mRNA and protein levels, up-regulate enzymatic activities of CYP3A4	Zhang et al. (2014b)
LS174T cells	20 µM				

## Toxicity

When evaluating the efficacy of ingredients, the toxicity and safety of them should be considered particularly (Sun et al., 2020b). For a long time, traditional Chinese medicine (TCM) is well known for its safety. But in recent years, the adverse reactions have been reported frequently. Being a diterpenoids compound broadly distributed in medicinal plants, oridonin has an extensive range of pharmacological activities. However, several lines of evidence indicated that oridonin may exhibit adverse effects, even toxicity under specific circumstances, which sparked intense debate and concern about security of oridonin. As discussed above, it was discovered that oridonin showed antitumor activity on small cell lung cancer (SCLC), but at the same time, HE staining revealed a certain degree of cytotoxicity in hepatic tissue after treatment with oridonin (10 mg/kg) (Xu et al., 2020). In addition, intervention of oridonin induced abnormalities in zebrafish, such as uninflated swim bladder and pericardial congestion at an EC_50_ of 411.94 mg/L *in vitro*, as well as it also decreased the body length of zebrafish. In this article, researcher relied on the fact that the downregulation of VEGFR3 gene expression probably be related to the occurrence of abnormalities following oridonin exposure during embryonic development (Tian et al., 2019). A 48 h exposure to oridonin (⩾25 µM) sharply augmented cytosolic Ca2^+^ concentration, potentiated formation of ceramide, and then triggered suicidal death of erythrocytes (Jilani et al., 2011).

On the other hand, some reports suggested that oridonin could induce the expression and activation of CYP2C and CYP3A family (Zhang Y. W. et al., 2018), and appeared to be a potential risk to herb-drug interactions as a result of its induction effects on drug processing genes expression and activation (Zhang Y.-w. et al., 2014). Therefore, these reports suggested that we should pay attention to the safety issues caused by the combination of oridonin in clinical practice. Generally speaking, there are few adverse reports on the safety of oridonin, but the lack of reports does not mean that there are no such potential risks. In view of this, it is particularly important to explore the mechanisms responsible for the adverse risk of oridonin under particular circumstances. Other toxicity researches of oridonin are shown in Table 3.

**TABLE 3:** Toxicity researches of oridonin.

Model	Dose	Detail	Ref
BALB/c mice	5–10 mg/kg	HE staining revealed a certain degree of cytotoxicity in hepatic tissue	Xu et al. (2020)
Zebrafish	100–400 mg/L	Decrease heartbeat with IC50 of 285.76 mg/L at 48 h, induce malformation at 120 h with half maximal effective concentration of 411.94 mg/L	Tian et al. (2019)
Erythrocytes	1 mM	Trigger Ca^2+^ entry and ceramide formation as well as suicidal death of erythrocytes	Jilani et al. (2011)
PXR-humanized mice	25–200 mg/kg	Induce the expression and activation of CYP2c and CYP3a family, which might contribute to potential drug–drug interactions and appear to be a risk when co-administered with other clinical drugs	Zhang et al. (2018b)
C57BL/6 mice	25–200 mg/kg	Appear to be a potential risk to herb-drug interactions as a result of its induction effects on drug processing genes expression and activation	Zhang et al. (2014b)

## Summary and Outlooks

Oridonin exists in considerable number of traditional herbal medicines and possesses salient medicinal value. Numerous researches have exhibited that it can regulate a variety of gene and protein expression such as ALP, IL-6, TNF-α, Bcl-2, caspase-3, PGE2, etc. It also shows extensive effects in the regulation of NF-*κ*B, PI3K/Akt/mTOR, and ERK1/2 signaling pathways. This review summarized the mechanism by which oridonin is utilized to treat related diseases (as shown in Table 1) and the related parameters of the pharmacokinetics (as shown in Table 2), as well as security problems in clinical practice (as shown in Table 3). However, there are some issues that need further clarification in future research.

Although oridonin has been proved to possess assorted pharmacological activities *in vivo* and *in vitro*, the specific mechanism of its biological activity has not been fully expounded. Hence, it is severely significant to further excavate the mechanism of pharmacological activity at molecular level.

Additionally, as described herein, it has shown prominent adverse effects, even toxicity under specific circumstances *in vitro* and *in vivo*. Hence, the conduction of essential investigations and comprehensive strategies to strike the balance between toxicological safety and therapeutic efficacy, as well as the establishment of an all-round research on the effect of dosage on pharmacological activity and toxicity, is highly demanded in this field.

As described herein, oridonin has shown prominent adverse effects, even toxicity under specific circumstances *in vitro* and *in vivo*. It showed hepatotoxicity and hepatoprotective effects, which the pair of pharmacological activities seems to be a paradox. However, through the analysis, it is found that this is mainly related to the concentration of oridonin and the time of administration. Long-term administration and high dose administration may cause liver damage. Therefore, it is necessary to further investigate the effects of the concentration of oridonin on pharmacological effects and toxicity. On the other hand, according to the chemical structure of oridonin, it may react covalently with the sulfhydryl group of some proteins, which can partly explain the reason of adverse reactions even toxicity of oridonin in specific environment. In addition, based on the analysis of the existing literatures, we think that the current researches are focus more on the toxicity of oridonin itself. Nevertheless, the toxic process of oridonin metabolites is still unknown. These aspects can be further interpreted in future. Therefore, in view of the above reasons for the safety of oridonin, we suggest that the conduction of essential investigations and comprehensive strategies to strike the balance between toxicological safety and therapeutic efficacy are necessary, as well as the establishment of an all-round research on the effect of dosage on pharmacological activity and toxicity, is highly demanded in this field.

**FIGURE 4:**
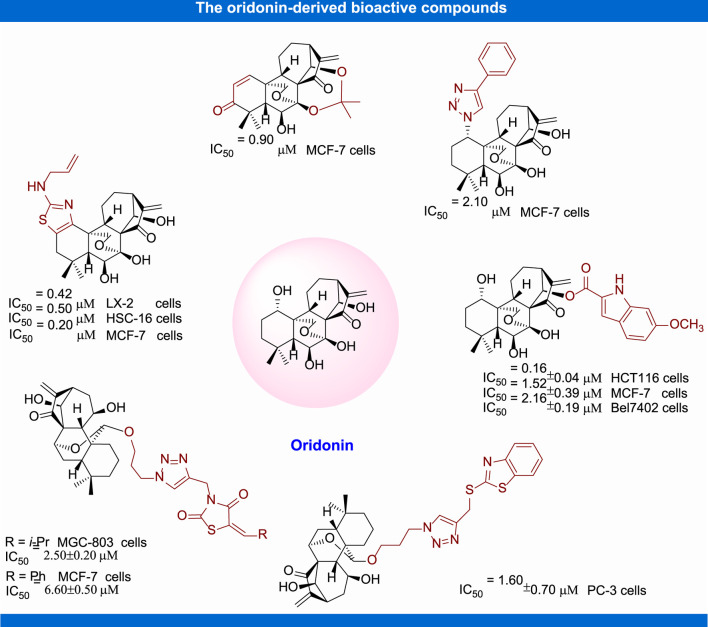
The structural modification of oridonin.

In recent years, structural modification of oridonin, including 1) the derivatization of hydroxyl groups, 2) modification of A-ring, 3) modification of the enone system, and 4) the transformation and derivatization of the framework structure, has been conducted in order to ameliorate the activity and amplify their application scope (Zhang et al., 2020). In the past decades, great progress has been made in structure activity relationship and mechanism of action studies of oridonin for the treatment of malignant tumor and other diseases (Figure 4). The structure and activity relation studies based on these new derivatives have tremendously contributed to the comprehension of their mechanism of actions and molecular targets.

According to the above literatures, we deeply realized that an increasing number of reports indicate that oridonin has miscellaneous positive pharmacological activities. However, on the whole, the oridonin’s specific mechanism related various diseases still remain to be clarified. On the other hand, although this natural active ingredient can positively influence the disease process by regulating multiple signal pathways or targets, it is only utilized as adjuvant agents in clinical practice, and rarely applied in the treatment of specific diseases. Therefore, in consideration of the current scattered research, detailed mechanism of oridonin in the treatment of specific diseases should be systematically integrated in the future.
